# Cooperation with rheumatologists on intensive systemic treatment for psoriatic arthritis-related panuveitis with retinal vasculitis: a case report

**DOI:** 10.1186/s12886-022-02313-1

**Published:** 2022-02-23

**Authors:** Po-Ying Wu, Chia-Chen Kao, Shwu-Jiuan Sheu

**Affiliations:** 1 Department of General Medicine, Kaohsiung Medical University Hospital, Kaohsiung Medical University, Kaohsiung, Taiwan; 2 Department of Ophthalmology, Kaohsiung Medical University Hospital, Kaohsiung Medical University, Kaohsiung, Taiwan; 3 School of Medicine, Kaohsiung Medical University, Kaohsiung, Taiwan

**Keywords:** Uveitis, Psoriasis, Psoriatic arthritis, Retinal vasculitis

## Abstract

**Background:**

Patients with psoriatic arthritis (PsA) may develop uveitis, a potentially serious ocular complication. PsA-related uveitis may result in significant morbidity and even vision loss if underdiagnosed or under-treated. We presented a case with long-standing recurrent uveitis and retinal vasculitis successfully managed by fortified systemic immunomodulators for systemic PsA.

**Case presentation:**

A 47-year-old woman was referred under the impression of acute anterior uveitis in her right eye in recent one month. Ocular examinations showed panuveitis in both eyes with intense vitreous opacity in her right eye. Fundus fluorescence angiography revealed retinal vasculitis in both eyes. Systemic surveys excluded the possibility of infection but showed an elevated inflammation marker. With intensive immunosuppressive treatment, inflammation resolved and the vision improved.

**Conclusion:**

Our case highlights not only the importance of intensified systemic therapy in treating PsA-related uveitis but the importance of multidisciplinary collaboration. Recurrent uveitis may be an indicator of disease activity prior to other inflammatory markers.

## Background

Patients with psoriasis or psoriatic arthritis (PsA) may develop uveitis, a potentially serious ocular complication [1, 2]. Psoriatic arthritis, the most common coexisting comorbidity in psoriasis, is a type of arthritis that affects some people with psoriasis [3]. There are many ocular complications in psoriasis, which may involve any structures in the eye from conjunctiva, cornea, sclera to uvea [4]. Uveitis affects 8% of the patient with psoriasis and even higher in the patients with psoriatic arthritis [2]. Presentation in psoriatic uveitis is usually bilateral, chronic, and severe. Undiagnosed and under-treated cases of psoriatic uveitis may cause significant morbidity and even vision loss. Compared with other autoimmune-related uveitis, uveitis in psoriasis or PsA has a higher proportion of posterior uveitis [4, 5]. Retinal complications of psoriatic uveitis such as macular edema and retinal vasculitis were also reported previously [6]. The treatment for psoriatic uveitis, in general, confines the local treatment, including topical steroids in any forms or local treatment of injection of targeted biologic agents in the acute phase [7, 8]. However, to our best knowledge, few previous reports emphasized the importance of intensive systemic treatment in psoriatic uveitis. Herein, we presented a case with long-standing recurrent uveitis with retinal vasculitis successfully managed by fortified systemic immunomodulators for psoriatic arthritis. Our case reminds the clinicians of systemic treatment for psoriatic uveitis. Recurrent uveitis may imply the recurrence of the underlying disease. Timely referral to a rheumatology expert may be of paramount importance.

## Case presentation

A 47-year-old woman was referred to our clinic by the local medical doctor because of recalcitrant recurrent anterior uveitis in her right eye for one month. She had suffered from similar complaints on and off in both eyes alternatively for about 10 years. Iritis was diagnosed then. Her condition usually improved after topical treatment. However, in this time attack, the symptoms were much more severe than before and her vision got worse even by the aggressive topical steroid. Her past medical history includes psoriatic arthritis with positive human leukocyte antigen B27 (HLA-B27), Sjogren’s syndrome, and hypothyroidism. Her psoriatic arthritis was newly diagnosed six months before the referral with the initial presentation of iritis and right buttock pain. Medication for her psoriatic arthritis was slowly titrated to cyclosporine 100 mg/day (about 1.72 mg/kg/day; her body weight was 58 kg) due to poor control under either leflunomide, hydroxychloroquine, or sulfasalazine. The latest inflammatory marker of erythrocyte sedimentation rate (ESR) was 42 mm/hr (normal range < 15 mm/hr in females, the patient’s baseline when diagnosed as PsA: 38 mm/hr), and high sensitivity C-reactive protein (hsCRP) was 6.74 mg/L. Those were examined seven weeks before this time event.

**Fig. 1:**
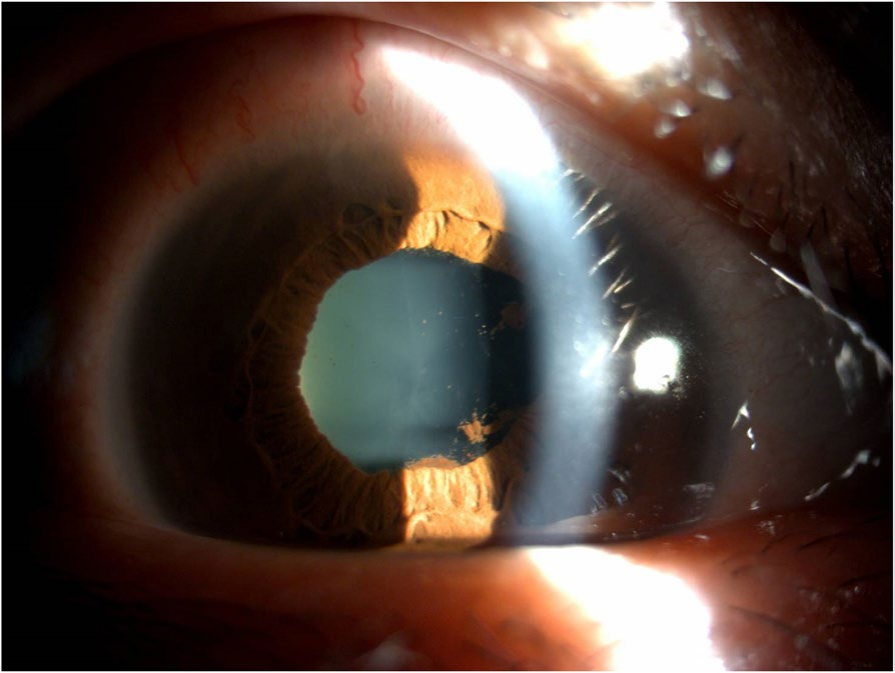
External eye photograph. Recurrent uveitis with anterior chamber intense reaction, posterior synechiae, and irregular pupil in the right eye the first time visiting our clinic

**Fig. 2:**
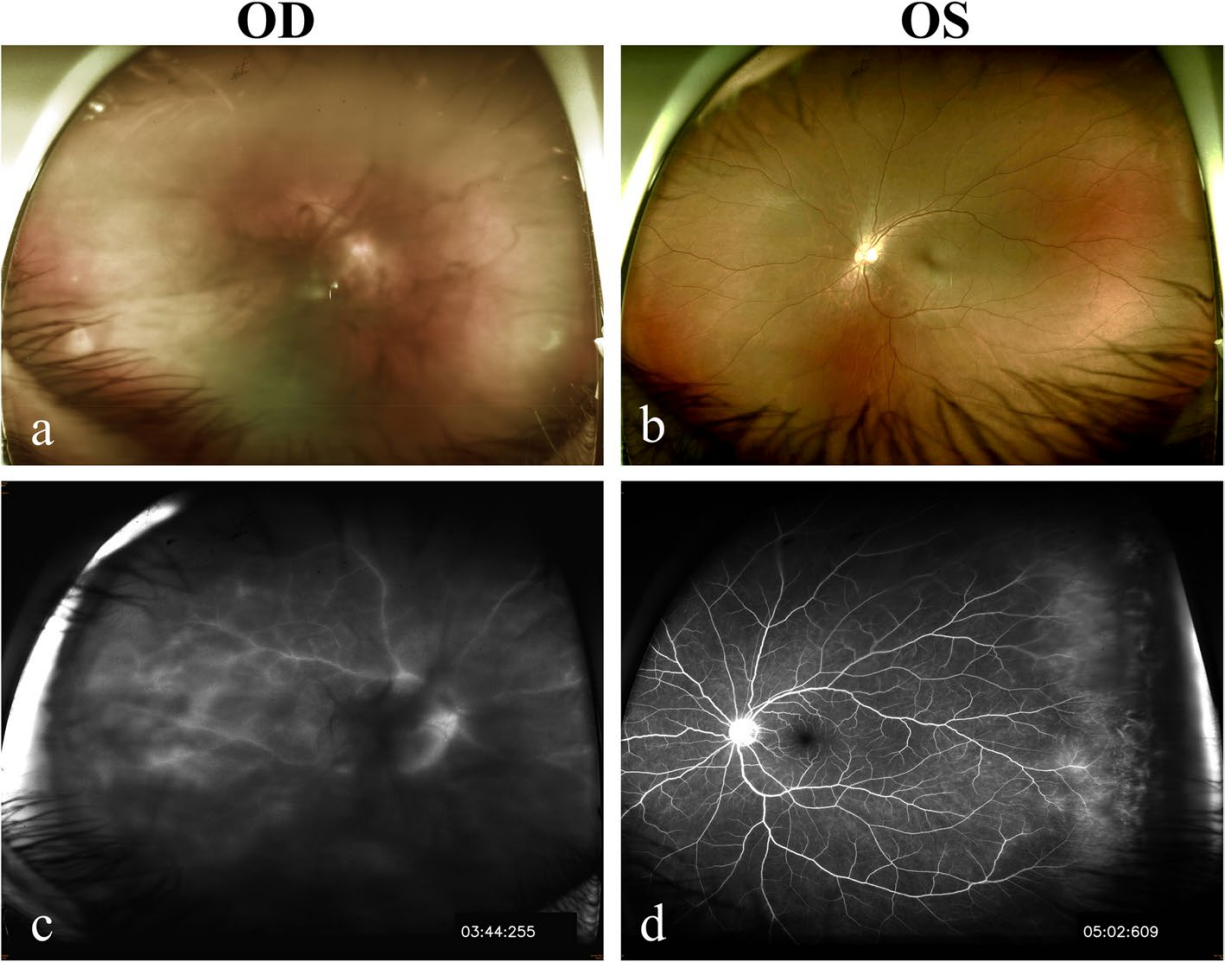
Fundus photographs and fundus fluorescein angiography (FA). The fundus photo showed severe vitreous opacity with 3 + haze severity according to the National Eye Institute classification in the right eye (**a**) and grossly normal in the left eye (**b**) initially. Though the image in the right eye from FA was mainly blocked by the dense vitreous opacity, peripheral vessel leakages and optic disc leakages were fairly visible (**c**). FA image in the left eye also showed peripheral vessel leakages (**d**)

On ocular examination, her best corrected visual acuity (BCVA) was 20/158 in the right eye and 20/20 in the left eye. Intraocular pressure (IOP) was normal with 10.4, and 12.3 mmHg in the right and left eye, respectively. Slit-lamp examination showed iritis, grade 3 + anterior chamber cells and posterior synechiae in the right eye (Fig. 1) and mild anterior chamber reaction (grade 1 + anterior chamber cells) in the left eye. Fundus showed severe vitreous opacity with 3 + haze severity in the right eye according to the National Eye Institute classification (Fig. 2a**).** Grossly normal fundus in the left eye was found (Fig. 2b). Optical Coherence Tomography (OCT) revealed no view of the right eye and mild vitreomacular adhesion in the left, and B scan of the right eye showed vitreous opacity but no retinal detachment. Fundus fluorescein angiography revealed retinal vasculitis in both eyes (Fig. 2c and d). Systemic surveys included chest x-ray, basic blood test, and virus antibodies, which excluded the possibility of infection; however, ESR at this time was elevated to 59 mm/hr; hsCRP was 8.06 mg/L.

**Fig. 3:**
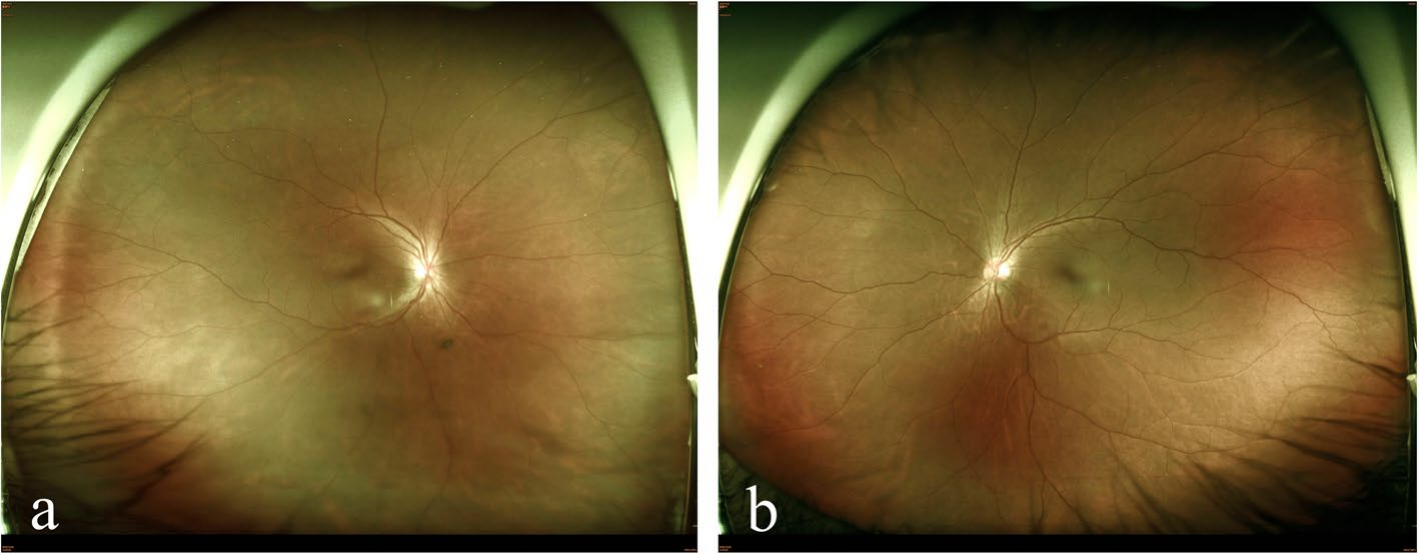
Fundus photographs. Three months after intensive treatment, the fundus showed grossly normal in the right eye (**a**) and the left eye (**b**)

**Fig. 4:**
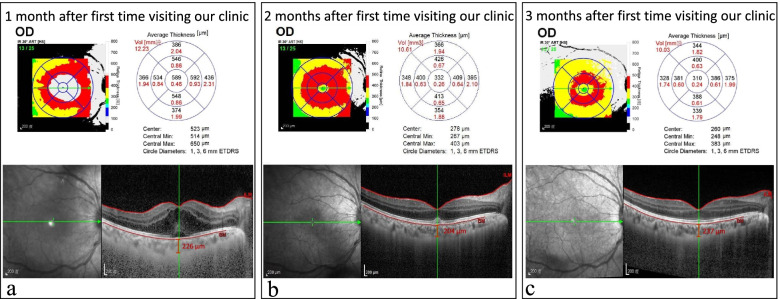
Images of Optical coherence tomography (OCT) in the right eye (RE) from 1 to 3 months after the first time visiting our clinic. Since there was severe vitreous opacity in RE initially, OCT revealed no view of RE when the first time visiting our clinic. One month after the first time visiting our clinic, OCT showed severe macula edema with central macular thickness of 589 μm and subretinal fluid was noted (**a**). Measured choroid thickness was 226 μm by Enhanced depth imaging OCT (EDI-OCT) (**a**). Two months after the first time visiting our clinic, the macula edema was decreased (332 μm) with minimal subretinal fluid in RE (**b**). Macular edema nearly resolved three months after first time visit (**c**)

Topical treatments including topical steroids for anti-inflammation and cycloplegia for posterior synechiae were kept for the right eye, but the inflammation worsened with more intense vitreous opacity. The patient was then referred back to her rheumatologist for intensified immunosuppressive treatment. Treatment for PsA was titrated to cyclosporine 125mg/day (2.16mg/kg/day) and methotrexate 10mg/week. The ocular inflammation resolved gradually. Three months after the event, her BCVA improved to 20/25 and 20/20 in the right and left eye, respectively. IOP remained normal with 14.8, and 13.6 mmHg in the right and left eye, respectively. The eyes were silent with clearly visible fundus in both eyes (Fig. 3a-b). Macula edema in the right eye was first detected after the vitreous haze decreased about one month after the first visit, and resolved gradually without additional subtenon or intravitreal steroid use (Fig. 4a-c). Following ESR and hsCRP were decreased to 41mm/hr and 6.43 mg/L, respectively, 3 months after the event. The patient was also regularly followed up in the rheumatologist with no major systemic side effects found after intensified immunosuppressive treatment. The eye remained silent except progressive lens opacity in the right eye.

## Discussion

Psoriasis and PsA are both immune-mediated diseases, which may coexist with plenty of comorbidities [1, 2]. Comorbidities ranged from mental illness, cardiovascular diseases, to broad ocular problems [9]. Therefore, multidisciplinary collaboration is of interest. Uveitis was initially thought to be related only to PsA since the incidence tends to be higher in patients suffering from PsA than in psoriasis only [5]. However, there is growing evidence showing the association independently between uveitis and psoriasis [6, 7]. PsA belongs to the spondyloarthropathy (SA), a group of disorders featuring enteritis and a high incidence of positive HLA-B27. SA-related uveitis shares some characteristics, most of which presents as acute anterior uveitis [8]. In comparison to other SA-related uveitis, uveitis in PsA is often more continuous, insidious in onset, posterior, and bilateral [5]. The similarity of these characteristics can also be found in psoriatic uveitis [7, 10].

Posterior uveitis may present as, for instance, macular edema and retinal vasculitis [5, 6]. Retinal vasculitis, involving the retinal vessels, is sight-threatening. Detection of retinal vasculitis can be made clinically and confirmed by the fundus fluorescein angiography. It may occur as various conditions and can be found in patients with SA-related uveitis [11]. The treatment for retinal vasculitis depends on the cause of the vasculitis and its severity. In our case, OCT was failed to perform in the right eye and the fundus examination seemed hard to examine due to vitreous opacity in the right eye but grossly normal in the left eye initially. Fundus fluorescein angiography showed peripheral vessel leakages in both eyes. This implied the routine fundus examination or OCT may not be sufficient once posterior uveitis is suspected.

The pathophysiology of psoriasis, PsA, and uveitis remains elusive. Some propose the activation of neutrophils [10] or T-cells, especially Th1/Th17 cells [7], plays a crucial role in the links with uveitis. Treatment for psoriatic uveitis differs according to either anterior or posterior uveitis happening. Standard therapy for acute anterior uveitis is topical corticosteroids [4]. Cycloplegic agents are needed to keep the pupil mobile and prevent the formation of synechiae. When posterior uveitis involves, the inflammation treatment is more urgent since posterior uveitis is more sight-threatening. With the understanding of the pathogenesis, specific inflammatory mediators have been identified and the use of drugs targeting the specific pathway has been suggested in more refractory cases [12].

Systemic treatment should be considered in refractory or posterior uveitis. Although the steroid is usually the first choice, patients with positive HLA-B27 are more likely to require systemic immunomodulation for the control and prophylaxis of ocular inflammation though HLA-B27 positivity is not associated with severity of inflammation [13]. The timing for referral to an internal expert may be at the time that local treatment fails or serious sight-threatening complications develop. It implies refractory diseases when patients are under high dose medication for underlying diseases. Simple local treatment may be insufficient. In our case, the inflammation worsens even under high-intense topical steroids in conjunction with cyclosporine, which implied the need for fortified systemic treatment. Elevation of ESR on the day of referral showed poor control for the underlying disease then. This also suggested the uveitis status was related to the flare up of the patient’s underlying PsA status. After the addition of methotrexate (10 mg/week) and application of a higher dose for cyclosporine (125 mg/day, 2.16 mg/kg/day), ocular and systemic inflammation resolved gradually and her visual acuity improved.

In conclusion, underlying diseases, especially autoimmune diseases, should be carefully managed in patients with uveitis and be cooperated with different specialists. Significant morbidities and even vision loss may happen in undiagnosed or under-treated psoriatic uveitis. Ocular inflammation and visual prognosis often improve after timely and adequate treatment. Our case supports the importance of both intensive systemic treatment and cooperation of multidiscipline in the management of PsA-related panuveitis with retinal vasculitis.

## Data Availability

The datasets used and/or analyzed during the current study are available from the corresponding author on reasonable request.

## Abbreviations

PsA: psoriatic arthritis
HLA-B27: human leukocyte antigen B27
ESR: erythrocyte sedimentation rate
CRP: C-reactive protein
BCVA: best corrected visual acuity
OCT: Optical Coherence Tomography
IOP: intraocular pressure
SA: spondyloarthropathy
